# Determinants of uncontrolled hypertension in adult type 2 diabetes mellitus: an analysis of the Malaysian diabetes registry 2009

**DOI:** 10.1186/1475-2840-11-54

**Published:** 2012-05-18

**Authors:** Boon How Chew, Ismail Mastura, Sazlina Shariff-Ghazali, Ping Yein Lee, Ai Theng Cheong, Zaiton Ahmad, Sri Wahyu Taher, Jamaiyah Haniff, Feisul Idzwan Mustapha, Mohd Adam Bujang

**Affiliations:** 1 Department of Family Medicine, Faculty of Medicine & Health Sciences, Universiti Putra Malaysia, 43400, Serdang, Selangor, Malaysia; 2 Klinik Kesihatan Seremban 2, Negeri Sembilan, Malaysia; 3 Klinik Kesihatan Bandar Sungai Petani, Kedah, Malaysia; 4 Clinical Research Centre, Hospital Kuala Lumpur, Kuala Lumpur, Malaysia; 5 Disease Control Division, Ministry of Health Malaysia, Putrajaya, Malaysia

**Keywords:** Type 2 Diabetes Mellitus, Hypertension, Antihypertensive agents, Primary care

## Abstract

**Background:**

Uncontrolled blood pressure (BP) is a significant contributor of morbidity and even mortality in type 2 diabetes (T2D) patients. This study was done to determine the significant determinants of uncontrolled blood pressure in T2D patients in Malaysia.

**Methods:**

Between 1^st^ January 2009 to 31^st^ December 2009, data from 70 889 patients with Type 2 diabetes was obtained from the Adult Diabetes Control and Management Registry for analysis; 303 centers participated in the study. Their demographic characteristics, the nature of their diabetes, their state of hypertension, treatment modalities, risk factors, and complications are described. Based on their most recent BP values, subjects were divided into controlled BP and uncontrolled BP and their clinical determinants compared. Independent determinants were identified using multivariate logistic regression.

**Results:**

The mean age of patients at diagnosis of diabetes was 52.3 ± 11.1 years old. Most were women (59.0 %) and of Malay ethnicity (61.9 %). The mean duration of diabetes was 5.9 ± 5.6 years. A total of 57.4 % were hypertensive. Of the 56 503 blood pressure (BP) measured, 13 280 (23.5 %) patients had BP <130/80 mmHg. Eighteen percent was on > two anti-hypertensive agents. Health clinics without doctor, older age (≥ 50 years old), shorter duration of diabetes (< 5 years), Malay, overweight were determinants for uncontrolled blood pressure (BP ≥130/80 mmHg). Patients who were on anti-hypertensive agent/s were 2.7 times more likely to have BP ≥130/80 mmHg. Type 2 diabetes patients who had ischaemic heart disease or nephropathy were about 20 % and 15 % more likely to have their blood pressure treated to target respectively.

**Conclusions:**

Major independent determinants of uncontrolled BP in our group of T2D patients were Malay ethnicity, older age, recent diagnosis of diabetes, overweight and follow-up at health clinics without a doctor and possibly the improper use of anti hypertensive agent. More effort, education and resources, especially in the primary health care centres are needed to improve hypertensive care among our patients with diabetes.

## Background

The profile of blood pressure (BP) management in Type 2 diabetes mellitus (T2D) patients has generally been unsatisfactory until about two decades ago [1,2]. In 1987, the Hypertension in Diabetes Study (HDS) revealed that most of the hypertensives went unrecognized, untreated and if treated, the target blood pressure (BP) was unacceptably high when compared to the current criteria [3]. The study also revealed the benefits of lowering blood pressure in these patients. It showed that the reduction of a mean blood pressure of between 5 to 10 mmHg, reduced the diabetes related deaths by one-third , the incidence of stroke by almost half and reduced the incidence of heart failure by almost one-third after a median follow-up period of 8.4 years. Furthermore, detailed analysis showed that the number needed to treat (NNT) to prevent one major complication of diabetes was 6 patients and 15 patients for death [3]. These benefits also appeared more favourable than those resulting from the intensified glycaemic control strategy for microvascular endpoints (NNT 138 vs 357). Many other studies had showed similar detrimental consequences of poor control of hypertension in diabetics. A systematic review of observational studies involving close to 48 000 patients showed that uncontrolled hypertension had a significant impact on diabetes-related complications [4]. Also, of importance was Framingham study which showed that while the risk of death (7 %) and cardiovascular events (9 %) could be attributed to diabetes, the risk of death and cardiovascular events attributed to co-existent hypertension were far higher; 44 % and 41 % respectively [5].

Patients with diabetes are especially vulnerable to hypertensive injury. The coexistence of hypertension has a significant impact of the poor prognosis for patients with diabetes because of its effect on the micro and macro vasculature. These include impaired autoregulation of blood flow in the microcirculation, the non-dipping of nocturnal BP owing to autonomic dysfunction, increased pulse-wave velocity and ventricular-vascular mis-coupling from premature stiffening of the abdominal aorta by elastic fibres glycation [6-9]. The additional advantages of intensive and good BP control for T2D patients are the increase of quality-adjusted life-years (QALYs) and cost-effectiveness. This benefit resulted from reduced cost of managing complications, increased survival and an increase in the interval-free complications [10,11]. Furthermore, the cost of managing a T2D patient is not cheap. In Malaysia even in the Public Health Service, the cost averaged around US 350 dollars a month if the patient saw a family physician or US 250 dollars if he or she saw a non-specialist. If the patient was admitted for treatment the cost doubled and , if for complications e.g. stroke, foot gangrene, the cost would escalate 10-fold [12].

Clearly there is convincing evidence on the benefits of effectively treating T2D patients who have hypertension. However, we have no national data on this aspect of the problem and neither do we know how effective we have been in dealing with the problem. We therefore set out to assess the care these patients have been given and in particular, the variables that had a significant impact on the uncontrolled blood pressure. We hoped that the results will shed some light on the problem and guide policy-makers in developing appropriate strategies for prevention and for the proper allocation of health resources to this area of diabetic management.

## Methods

This was a cross-sectional study using data extracted from the Adult Diabetes Control and Management (ADCM) Registry in 2009. It represents 70 889 T2D patients from 289 health clinics and 14 hospitals and from 8 of the 15 states in the country and 2 Federal territories. These represent 22.1 % of the health clinics and 9.6 % of the hospitals in the country. It has been estimated that these patients represent 5.2 % of the total number of diabetics in these areas (70889/1368590) [13]. Up to 31^st^ December 2009, 3140 (4.4 %) were lost to follow-up (defaulted appointment for > one year) and 203 (0.3 %) patients had passed on.

Only adult patients (≥18 years of age) were registered. All patients were informed of the on-going registry and given the opportunity to opt out. However, the participation in ADCM was non-mandatory for patients and health centres. Data collection at local centers was performed by trained physicians, assistant physicians and nurses. All data on the participating patients were registered on an on-line standard case record form (CRF) and the information was made available in the ADCM website. This website was maintained by Clinical Research Centre (CRC), Ministry of Health, Malaysia.

### The Malaysian health care system for patients with diabetes

The health care system in Malaysia is supported by the public and the private sectors [14]. The private health care provides about two thirds of the country’s medical specialists and caters for about one third of the upper-middle income groups of the population [15]. The public health care is organized by Ministry of Health and structured into the public health clinics and hospital care. The health clinics are well-linked to the secondary and tertiary public hospitals with its unique referral system. The patients in health clinics generally are managed by family medicine specialists (FMS), medical and health officer (M&HO), physician assistant and supported by specialized nurses and dietitians/nutritionists. In hospitals, care is provided by specialists in internal medicine or endocrinology, medical officers and specialized nurses. Under the system, every patient diagnosed with diabetes mellitus will receive a green booklet which will be kept by the patient. This is accompanied with a bigger green medical record book (kept at the health centre) that records all information pertaining to the care provided for the patient’s management. Those patients managed in the health clinics and who have complications are referred to the hospitals either for admission or for a co-shared care as outpatients. In health clinic without a FMS/M&HO, the cases are normally managed by physician assistants. Nurses at these health clinics contributed in measuring clinical parameters during patients’ visit and giving health education at the appointment day. Another reason for this referral is for the prescription of more expensive anti-diabetic agents (ADA), lipid-lowering agents or the newer or more expensive anti-hypertensive drugs. These are restricted items and can only be dispensed at the hospitals [16]. All major classes of anti-hypertensive drugs (including renin inhibitors, angiotensin receptor blockers, calcium channel blockers) are available in the public health sector. The newer, original and more expensive drugs are restricted and those that of older and generic are commonly used in patient care in compliance to the national clinical practice guideline.

### Definitions of study participants

A person was considered to have T2D if there was documented evidence of a diagnosis of diabetes mellitus (WHO criteria) and who was being treated either by life-style modification, oral ADA or insulin. Hypertension was diagnosed if the systolic blood pressure was ≥130 mm Hg or the diastolic blood pressure was ≥ 80 mm Hg on each of two successive readings measured in rested position with arm at heart level using a cuff of appropriate size; a BP < 130/80 mmHg was regarded as well controlled. Body mass index (BMI) was calculated as weight (kilogram) divided by height (metre) squared and categorized into underweight (<18.5), normal (18.5-22.9) and overweight (≥23.0); for the category of obesity it was further classified into pre-obese (23.0-27.4), obese I (27.5-34.9), obese II (35.0-39.9) and obese III (≥ 40). For their lipid profiles, a low density lipoprotein-cholesterol (LDL-C) ≤ 2.6 mmol/L, triglyceride (TG) ≤ 1.7 mmol/L and high density lipoprotein-cholesterol (HDL-C) ≥ 1.1 mmol/L were regarded as good treatment targets [17].

A diagnosis of cerebrovascular disease/stroke (CVD), ischemic heart disease (IHD), retinopathy, nephropathy and foot problem were based on symptoms, signs, laboratory results, radiological evidence and treatment history. Nephropathy was diagnosed by the presence on ≥ 2 occasions and at least three months apart of any of the following: microalbuminuria, proteinuria, serum creatinine > 150 mmol/L or estimated glomerular filtration rate < 60mls/min (Cockroft-Gault formula). Foot problems were defined as any deformity resulted from ulcers, amputation, peripheral neuropathy or peripheral vascular disease.

### Statistical analysis

The independent variables of interest were the types of clinic and how it was manned, gender, ethnicity, age, duration of diabetes, body mass index (BMI), waist circumference, CVD, IHD, retinopathy, nephropathy, erectile dysfunction (ED), diabetic foot problems, anti-hypertensive treatment, glycaemic control [HbA1c ≤ 6.5 % (48 mmol/mol)] and lipid profile control (LDL-C ≤ 2.6 mmol/L, TG ≤ 1.7 mmol/L, HDL-C ≥ 1.1 mmol/L).

Mean levels were compared using the Student’s t test for unpaired samples and proportions compared with Chi square test. The normality of each variable was first tested by histogram and confirmed by Kolmogorov-Smirnov test. A two-tailed P value of < 0.05 was considered to be significant.

Having grouped the patients into controlled and uncontrolled BP using the latest mean BP values, logistic regression was used to determine the significant clinical determinants. The significant determinants were then identified and fitted into a multivariate logistic regression model using the stepwise method with uncontrolled BP as the dependent variable. Multicolinearity between the variables were checked with correlation matrix and inspected for the magnitude of the standard error (SE). None of the variables correlated with each other, r < 0.2 and SEs were all within 0.001 to 5.0. All data was analysed using STATA version 9.

## Results

Of the 70 889 patients with T2D analysed, most were women (59.0 %) and Malays 62 %) (Table 1). The mean age at diagnosis of diabetes was 52.3 ± 11.1 years and the mean duration of diabetes was 5.9 ± 5.6 years. The mean systolic and diastolic blood pressures (BP) were 136.7 ± 19.5 mmHg (95 % CI ,136.6 to 136.9) and 78.8 ± 10.6 mmHg (95%CI , 78.7 to 78.9) respectively. A total of 40 659 (57.4 %) patients were reported to be hypertensive. Of the 56 503 BP measurements recorded from these patients, 33.8 % had systolic BP < 130 mmHg, 44.8 % had diastolic BP < 80 mmHg and 23.5 % patients had BP <130/80 mmHg. A more detailed report on this has been published elsewhere [13].

**Table 1:** **Association of registered variables with blood pressure controlled to target, n = 56503**^**‡**^

**Variable**	**Total n (% of total)**	**Controlled BP n (%)**	**Uncontrolled BP n (%)**	**Test statistic value, p-value**
**Total**	56503 (100)	13280 (23.5)	43223 (76.5)	NA
**Type of Health Clinic, n = 53941**				
Without FMS/doctor	12009 (22.3)	2558 (21.3)	9451 (78.7)	48.81, < 0.001*
With FMS/doctor	41932 (77.7)	10221 (24.4)	31711 (75.6)	
**Gender**				2.66, 0.27*
Male	22548 (39.9)	5326 (23.6)	17222 (76.4)	
Female	33866 (59.9)	7927 (23.4)	25939 (76.6)	
Missing	89 (0.2)	27 (30.3)	62 (69.7)	
**Ethnicity**				344.98, <0.001*
Malay	35181 (62.3)	7484 (21.3)	27697 (78.7)	
Chinese	11051 (19.6)	2761 (25.0)	8290 (75.0)	
Indian	9627 (17.0)	2891 (30.0)	6736 (70.0)	
Others	558 (1.0)	119 (21.3)	439 (78.7)	
Missing	86 (0.2)	25 (29.1)	61 (70.9)	
**Age group (year)**				
Mean (SD)	(58.32, 11.27)	(57.21, 11.43)	(58.38, 10.86)	10.717, <0.001^#^
<30	445 (0.8)	147 (33.0)	298 (67.0)	143.83, <0.001*
30-49	11452 (20.3)	3128 (27.3)	8324 (72.7)	
50-69	35877 (63.5)	8029 (22.4)	27848 (77.6)	
> = 70	8729 (15.4)	1976 (22.6)	6753 (77.4)	
**Duration of diabetes (year)**				
Mean (SD)	(5.86, 5.56)	(5.99, 5.59)	(5.75, 5.47)	4.457, <0.001^#^
<5	28704 (50.8)	6555 (22.8)	22149 (77.2)	17.29, 0.001*
5-10	19528 (34.6)	4677 (23.9)	14851 (76.1)	
>10	7962 (14.1)	1979 (24.8)	5983 (75.2)	
Missing	309 (0.5)	69 (22.3)	240 (77.7)	
**BMI (kg/m2)**	53687 (100)	12677 (23.6)	41010 (76.4)	
Mean (SD)	(27.28,5.96)	(26.46, 5.16)	(27.53, 6.16)	17.72, <0.001^#^
Underweight < 18.5	890 (1.7)	317 (35.6)	573 (64.4)	250.44, <0.001*
Normal 18.5-22.9	8792 (16.4)	2544 (28.9)	6248 (71.1)	
Overweight ≥ 23.0	44005 (77.9)	9816 (22.3)	34189 (77.7)	
Pre-obese 23.0-27.4	20868 (38.9)	5118 (24.5)	15750 (75.5)	148.07, <0.001*
Obese I 27.5-34.9	19343 (36.0)	4065 (21.0)	15278 (79.0)	
Obese II 35.0-39.9	2815 (5.2)	478 (17.0)	2337 (83.0)	
Obese III ≥ 40	979 (1.8)	155 (15.8)	824 (84.2)	
**HbA1c** ≤ 6.5 % (48 mmol/mol)	26052 (46.1)	6228 (23.9)	19824 (76.1)	0.04
> 6.5 % (48 mmol/mol)	30451 (53.9)	7052 (23.2)	23399 (76.8)	
**LDL-C** ≤ 2.6 mmol/L	11990 (21.2)	3016 (25.2)	8974 (74.8)	<0.001
> 2.6 mmol/L	44513 (78.8)	10264 (23.1)	34249 (76.9)	
**TG, n = 44121**				<0.001
≤ 1.7 mmol/L	23867 (54.1)	6018 (25.2)	17849 (74.8)	
> 1.7 mmol/L	20254 (45.9)	4412 (21.8)	15842 (78.2)	
**HDL-C** ≥ 1.1 mmol/L	43986 (77.8)	10235 (23.3)	33751 (76.7)	0.01
> 1.1 mmol/L	12517 (22.2)	3045 (24.3)	9472 (75.7)	

**Figure 1:**
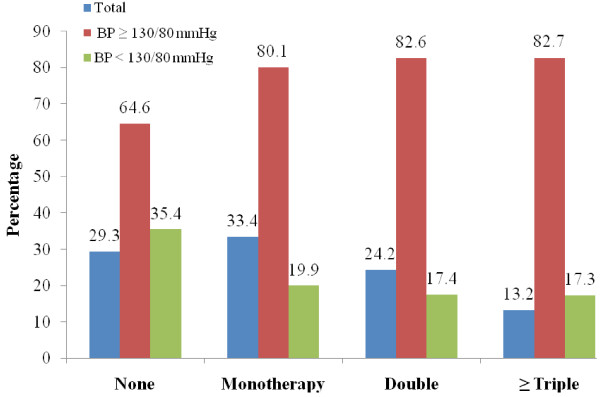
**Proportion of Type 2 Diabetes Patients Based on the Number of Blood Pressure Measurements (N = 56503) and Proportion of Blood Pressure Target Achieved According to the Number of Anti-hypertensive Agents.** A BP < 130/80 mmHg was regarded as controlled BP.

The variables that determined good control of BP on univariate analysis were, the type of health clinic, ethnicity, age group, duration of diabetes, BMI, HbA1c, targets of lipid profiles and use of anti-hypertensive agent (Table 1; Figure 1) Although insulin use was associated with blood pressures being controlled to target (χ^2^ = 19.14, p < 0.001), this association was not seen for ADA, diet therapy and HbA1c levels.

Of interest was that those who were being treated for hypertension did not have their blood pressure control on target (Figure 1) This was irrespective of whether they were on single (80.1 % vs 74.7 %, χ ^2^ = 204.66, p < 0.001), double (82.6 % vs 74.6 %, χ ^2^ = 371.69, p < 0.001) or ≥ triple anti-hypertensive agents (82.7 % vs 75.5 %, χ ^2^ = 186.98, p < 0.001).

Of the 41 286 (58.2 %) patients on anti-hypertensive agents, angiotensin-converting enzyme inhibitors (ACEI) (63.9 %) were the most prescribed, followed by calcium channel blockers (CCB) (37 %) beta-blockers (36.5 %) and diuretics (23.4 %). The use of specific anti-hypertensive drug classes had been assessed and reported elsewhere [13].

Health clinics without a doctor, older age (≥ 50 years old), shorter duration of diabetes (< 5 years), non-Indians (being Malay), overweight, being on anti-hypertensive agents and having poor lipid profile (LDL-C > 2.6 mmom/L) were predictors for uncontrolled blood pressure (BP ≥130/80 mmHg). Patients who were on anti-hypertensive agent/s were 2.7 times more likely to have uncontrolled BP. T2D patients who had IHD or nephropathy were about 20 % and 15 % more likely to have their blood pressure treated to target, respectively (Table 2).

**Table 2:** Multivariate logistic regression used stepwise method conducted for uncontrolled hypertension, n = 43223

**Independent variables:**	**OR**	**95 % CI**	**P value**
**Type of Health Clinic**			
Without FMS/doctor	1.11	1.04, 1.18	0.002
With FMS/doctor	1	-	-
**Age (years)**			
< 30	1	-	-
30-49	1.18	0.89, 1.56	0.24
50-69	1.30	0.99, 1.72	0.06
≥ 70	1.28	0.96, 1.71	0.09
**Ethnicity**			
Malay	1.41	1.30, 1.52	<0.001
Chinese	1.19	1.09, 1.31	<0.001
Indian	1	-	-
**Duration of diabetes (years)**			
< 5	1.12	1.02, 1.22	0.01
5-9	0.96	0.87, 1.05	0.33
≥ 10	1	-	-
**BMI (kg/m2)**			
< 23	1	-	-
≥ 23	1.35	1.26, 1.44	<0.001
**Anti-Hypertensive Agent**			
On Anti-Hypertensive/s	1	-	-
No Anti-Hypertensive	0.37	0.34, 0.39	<0.001
**Ischaemic Heart Disease**			
Absent	1	-	-
Present	0.84	0.72, 0.97	0.02
**Nephropathy**			
Absent	1	-	-
Present	0.87	0.78, 0.97	0.01
**LDL** > 2.6 mmol/L	1.13	1.06, 1.21	<0.001
≤ 2.6 mmol/L	1	-	-

CI = confidence interval; OR = odds ratio.

## Discussion

We set out to examine the status of our T2D patients with hypertension. The results were sub-optimal; less than one in four patients had their BPs controlled to the recommended target. This is despite convincing evidence that aggressive lowering of BP in people with diabetes reduces cardiovascular morbidity and mortality [18] Furthermore, the poor control persisted despite the fact that they were being treated and in some, with more than two antihypertensive agents. Others have had a similar experience [4,19-22].

Our findings suggested that treating BP to target was associated with less proportion of lipid profiles and HbA1c not at treatment targets (20-25 %). It was not the case in reverse when treating these lipid profiles and HbA1c to targets, we noticed that a large proportion of T2D patients were still not at BP target (about 75 %). Therefore, simultaneous management of all these risk factors is most cost-effective via BP lowering effort. Sever P et al reported a potential synergy between the use of amlodipine-based blood pressure lowering agent and lipid lowering agent (atorvastatin 10 mg) in the Anglo-Scandinavian Cardiac Outcomes Trial (ASCOT) which comprised 10 305 patients with about a quarter of them were with diabetes mellitus [23]. There was a 53 % reduction in the relative risk of coronary heart disease when patients received both these agents compared to using only statins (36 %). Perhaps, as has been confirmed by other studies, such combinations should be started early in our patients in order to achieve maximum benefit [24,25].

Of the demographic characteristics, we found that those who were > 50 years old, recently diagnosed with diabetes, overweight (and obese) and of Malay ethnicity were more likely to have uncontrolled BP and require closer monitoring. The high proportion of Malays was probably a reflection of the population distribution in Malaysia with Malays being the dominant race. It has been shown that a combination of T2D, resistant hypertension and obesity was related to autonomic imbalance (heart rate variability) and circadian disruption of the sympathetic and parasympathetic tones during day and night periods. These effects may contribute to the observed increased risk of cardiovascular morbidity and mortality [26]. Preventive measures should therefore concentrate on these patients. In our experience, gender was not an independent risk factor for uncontrolled BP. The Western experience was different. In Sweden, female patients with diabetes were found to exhibit more uncontrolled hypertension [27]. The Mayo clinic experience in the USA showed that in their group of 1090 diabetic patients with hypertension, older female, with isolated systolic hypertension and with uncontrolled BP at baseline were predictors of uncontrolled BP (≥ 130/85 mmHg) [28]. Our patients who had either nephropathy or IHD had better control of their blood pressures. The experience in MAYO clinic was similar [28]. The most probable reason was that these patients were under the care of specialists and in the hospitals. However, the Swedish experience was different; despite more than 94 % of them being on anti-hypertensives, good control of blood pressure was achieved in only 60 % of these patients [29].

The way in which our patients were being treated with hypertensive agents appears to be less than satisfactory. More than half of the patients were on two or more drugs and despite this; good control has not been achieved. Granted that most patients with T2D diabetes may require combination therapy [30] but the proper combination is important. The combination of a renin-angiotensin-aldosterone system (RAAS) inhibitors and a CCB has been shown to be an effective combination [31,32].

Therefore the education of the physicians managing these patients, the use of proper combinations, attending to the individual needs of the patient and adherence to guidelines are ways in which this area can be improved [33]. Thus, training program in this aspect of prescribing for diabetic hypertensive is ever more needed for the majority non-specialist primary care physicians. Pertinent aspects of drugs prescription should include safety issues such as monitoring of renal function post-initiation of a RAAS inhibitor, not over-aggressive in treating systolic BP (< 120 mmHg) in T2D patients who are at high risk for CVD events [34]. Patient-centred consultation strategies to enhance patient participation in decision making and to empower patient on hypertension self-management that would improve therapy adherence [35]. Besides, the clinicians should be encouraged out of inertia and taught on adding the third and more anti-hypertensive agents judiciously because some over-weighing the risk of polypharmacy against the optimal BP control [36]. The limited availability of certain anti-hypertensive agents such as the long-acting CCB and ARB could be the underlying cause of the majority patients under non-specialist primary care physician care did not achieve target BP level.

Not surprisingly, BP control among the T2D patients was better at the health clinics where a doctor was present when compared to those without. Rohana D et al has shown convincing evidence that in Malaysia, where the clinics were manned by a M&HO or family physician, the care was significantly better [37]. The solution therefore is to man more clinics with doctors or if that is not possible, have in place a proper and appropriate referral system for those patients whose hypertension prove difficult to control. An alternative is to transfer these patients to a neighbouring general practice clinic but the organization and logistics of doing so can be difficult [38].

There may be other factors that may have contributed to the poor control of hypertension. Concentration on the glycaemic control (HbA1c) at the expense of the hypertension could be one factor [39,40]. Inadequate training for our physicians on the principles of evidence based guidelines and health system that support these guidelines adherence may be another [41-43]. Failure to individualise treatment according to the needs of the patient and the lack of a team approach involving nurses, medical assistants, pharmacists, nutritionists/dietitians may be another factor [40,44,45]. Providing guidelines, having appropriate protocols and knowledge instruments that has been validated for use in this country should be put in place and then, perhaps the medical care for diabetic hypertensive would improve [42,46].

The limitations of our study are recognized. The sample studied may not have been representative of the country. Not all hospitals or health clinics were involved in the survey. Furthermore, 20 % of the blood pressure recordings were missing [47]. A large proportion of diabetic patients are being managed in the private sector and the situation with regards to control of blood pressure may have been different. Other factors that may affect the results such as the socioeconomic status, health literacy, professional support, mental disorders, smoking, physical activity, alcohol consumption, medication adherence etc. were not considered in our study [48,49]. Notwithstanding these deficiencies, we feel that a large sample size of 70,000 patients and from their demographic characteristics may actually be representative of patients with T2D seeking treatment in the Public Health Centres.

## Conclusions

T2D patients who were older, recently diagnosed, obese and having care at a health clinic without a doctor were more likely to have uncontrolled BP. These patients can be clearly identified and therefore preventive measures should concentrate on this group of patients. The inappropriate use of anti-hypertensive agent may be another identifiable reason for the poor control of hypertension. Investment in these areas in terms of appointment of trained physicians to health clinics and drawing up proper guidelines in their management of hypertension, especially in improving physician prescribing capability, should be a priority in order to reduce the unfavourable consequences of hypertension in our patients with T2D.

## Abbreviations

T2D, Type 2 diabetes; BP, Blood pressure; HDS, Hypertension in Diabetes Study; NNT, Number needed to treat; QALYs, Quality-adjusted life-years; ADCM, Adult Diabetes Control and Management; CRF, Case record form; FMS, Family medicine specialists; ADA, Anti-hyperglycaemic agents; LDL-C, Low density lipoprotein-cholesterol; TG, Triglyceride; HDL-C, High density lipoprotein-cholesterol; CVD, Cerebrovascular disease/stroke; IHD, Ischemic heart disease; ED, Erectile dysfunction; BMI, Body mass index; ACEI, Angiotensin-converting enzyme inhibitors; CCB, Calcium channel blockers.

## Competing interests

The authors declare that they have no competing interests.

## Authors’ contributions

MI & CBH collected data; CBH drafted the manuscript; MAB & CBH, performed statistical analysis. All authors read, edited and approved the final manuscript.
